# *De novo* [*PSI*^+^] prion formation involves multiple pathways to form infectious oligomers

**DOI:** 10.1038/s41598-017-00135-6

**Published:** 2017-03-06

**Authors:** Jaya Sharma, Brett T. Wisniewski, Emily Paulson, Joanna O. Obaoye, Stephen J. Merrill, Anita L. Manogaran

**Affiliations:** 1 Department of Biological Sciences, Marquette University, Milwaukee, WI 53201 USA; 2 Department of Mathematics, Statistics and Computer Science, Marquette University, Milwaukee, WI 53201 USA

## Abstract

Prion and other neurodegenerative diseases are associated with misfolded protein assemblies called amyloid. Research has begun to uncover common mechanisms underlying transmission of amyloids, yet how amyloids form *in* *vivo* is still unclear. Here, we take advantage of the yeast prion, [*PSI* ^+^]*,* to uncover the early steps of amyloid formation *in vivo.* [*PSI* ^+^] is the prion form of the Sup35 protein. While [*PSI* ^+^] formation is quite rare, the prion can be greatly induced by overexpression of the prion domain of the Sup35 protein. This *de novo* induction of [*PSI* ^+^] shows the appearance of fluorescent cytoplasmic rings when the prion domain is fused with GFP. Our current work shows that *de novo* induction is more complex than previously thought. Using 4D live cell imaging, we observed that fluorescent structures are formed by four different pathways to yield [*PSI* ^+^] cells. Biochemical analysis of *de novo* induced cultures indicates that newly formed SDS resistant oligomers change in size over time and lysates made from *de novo* induced cultures are able to convert [*psi* ^−^] cells to [*PSI* ^+^] cells. Taken together, our findings suggest that newly formed prion oligomers are infectious.

## Introduction

Prions are self-perpetuating amyloids comprised of misfolded proteins. These misfolded proteins are able to convert normally folded versions to the misfolded assembled form that is infectious. In humans, prions are associated with the fatal Creutzfeldt-Jakob disease^1, 2^. While other human amyloid diseases exist, such as Alzheimer’s disease, Parkinson’s disease and Amyloidosis transthyretin (ATTR), prion disease was originally set apart because of its infectious nature^3, 4^. More recently, work from several labs has provided evidence that many amyloid diseases have prion-like qualities^5–10^ and have many more commonalities beyond protein aggregation, such as the existence of variant structural strains^11–16^ and the ability to cross seed from pre-existing amyloids (heterologous cross seeding)^17^. These commonalities suggest that knowledge gained regarding one amyloid can provide insight into the others.

Our understanding of prion-like behavior has been dramatically enhanced through the study of yeast prions^18^. Yeast prion aggregates are similar to their mammalian counterparts in that they form amyloid, are proteinase K resistant, exist as structural variants or strains, and can undergo heterologous cross seeding^18^. While several yeast prions have been identified, we have learned the most from the [*PSI* ^+^] prion. The [*PSI* ^+^] prion is the misfolded, amyloid form of the translation termination factor, Sup35p^19, 20^. The protein only hypothesis, in which proteins can act as infectious agents, was first proven with [*PSI* ^+^], since the introduction of recombinant Sup35p aggregates leads to [*psi* ^−^] cells to becoming [*PSI* ^+^]^21, 22^. The propagation of [*PSI* ^+^] is dependent on several chaperones including Hsp104p that mediate propagation by shearing large prion aggregates into heritable prion seeds, which are called propagons^20, 23–29^.

While much is known about prion propagation, the process of prion formation is less understood. The spontaneous frequency of prion formation is extremely low^30–33^. Overexpression of Sup35p increases the frequency of [*PSI* ^+^] formation^34^, presumably because the greater number of protein molecules present makes it more likely that a small portion of proteins misfold and form prions^19^. This *de novo* induction of prion formation is dramatically enhanced by the presence of another prion called [*PIN* ^+^], or [*RNQ* ^+^], which is the misfolded prion form of the Rnq1 protein^35–38^. While several hypotheses have been proposed to elucidate the role of [*PIN* ^+^] in [*PSI* ^+^] formation, the well-supported ‘cross seeding model’ suggests that the [*PIN* ^+^] prion acts as a template to cross seed [*PSI* ^+^] during the early stages of prion formation^35, 39, 40^. Once [*PSI* ^+^] is propagated, [*PIN* ^+^] can be lost without affecting further [*PSI* ^+^] maintenance.

The N-terminus of Sup35p is sufficient for the formation of the prion^41, 42^. The N-terminus and middle domains, when fused to a fluorescent tag, can join pre-existing prion particles^43^, and allow for the visualization of prion formation^44^. During *de novo* induction, small fluorescent foci initially form^40^. It has been proposed that these foci eventually develop into a ring or line structure located peripherally in the cytoplasm^40^. These ring or line-like aggregates are considered a hallmark of [*PSI* ^+^] formation because isolation of ring containing cells gives rise to [*PSI* ^+^] progeny, but sibling cells lacking any aggregates do not^45^. After cell division, the mother retains the ring and the daughter cell inherits sheared prion seeds that can propagate the [*PSI* ^+^] prion^46^.

Little is known about the early stages of prion formation. Several studies have used periodic “snapshots” to extrapolate the events associated with *de novo* prion induction, but there is no study to date that characterizes how new prion complexes are formed and whether they are infectious. We have found that the *de novo* induction of [*PSI* ^+^] can proceed via several different pathways. This induction involves the formation of SDS resistant oligomers that change in size over time. We also show, for the first time, that lysates containing newly formed oligomers have infective potential, suggesting that these developing oligomers may be involved in prion conversion.

## Results

### 4D live cell imaging reveals multiple pathways of *de novo* [*PSI*^+^] induction

**Figure 1:**
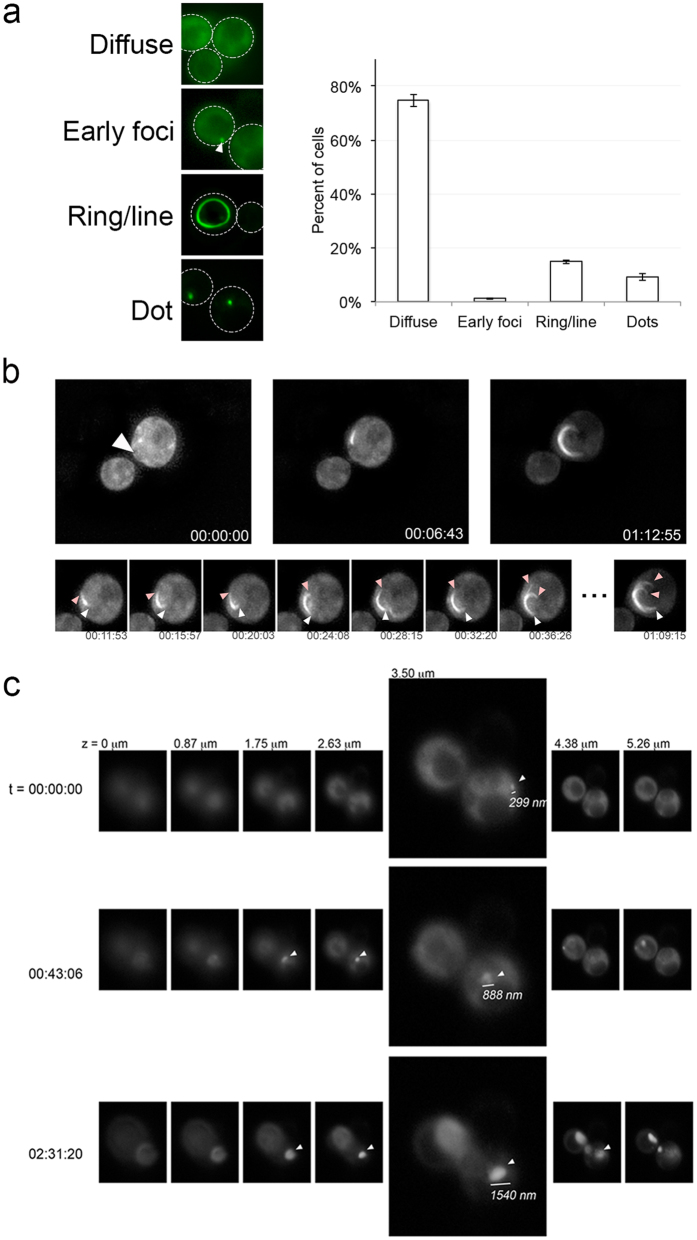
Formation of fluorescent structures begins as an early focus and develops into line and dot structures with distinctive growth patterns. (**a**) Sup35PrD-GFP was overexpressed in [*psi* ^−^][*PIN* ^+^] strains for approximately 18 hours. Cells were categorized as having diffuse cytoplasmic fluorescence (diffuse), the presence of a small focus/several foci on a diffuse background (early foci; indicated with arrow), rings/lines, or dots. The percentage of cells containing each fluorescent structure was determined by counting more than 700 cells from 3 independent transformants. Standard error is shown. (**b**) After 18 hours of overexpression, diffuse cells were visualized for an additional 12 hours using 3D time-lapse microscopy. Early foci (left top panel, arrow), mid (middle panel), and late stages (right panel) of ring formation (time shown as HH:MM:SS) relative to the first appearance of early focus are shown. The lower panel shows a more detailed time-lapse, with different colored arrows (white and pink) indicating different ends. All images shown are maximum projection. (**c**) Z-stacks (indicated as μm), corresponding to specific time points (rows), is shown for a single cell forming a single focus over time. 3.5 μm is expanded for more detail. The diameter of the growing dot structure was measured, as indicated by representative line. Arrows indicate when the focus is visible in each z-stack.

Previous characterization of fluorescent structures during the *de novo* induction pathway has focused on capturing images of cell populations at different time points, or isolating individual cells and imaging them periodically until they form microcolonies^40, 44–47^. We took a 4D live cell imaging approach where 3D images were captured over time in order to visualize continuous induction *in vivo*. Overnight expression of Sup35PrD-GFP, which contains the N-terminal and middle domain of Sup35p, gave rise to approximately one quarter of GFP expressing cells containing fluorescent structures using a *CUP1* promoter (Fig. 1a). These fluorescent structures included cells with small foci in diffuse GFP backgrounds, rings, lines, and dots (Fig. 1a). In order to follow the entire fluorescent structure formation process, we observed overnight induced cell cultures microscopically. Cultures were induced to express Sup35PrD-GFP for approximately 18 hours. Cells were followed for an additional 6–12 hours under time-lapse. We examined in excess of 2000 cells. Of those cells, 382 had ring, line or dot structures at the end of time-lapse. We were able to capture the complete aggregate formation process in 92 cells, where the beginning of fluorescent structure formation could be visualized. We observed that all cells start with one to several transient foci on a diffuse background, which we call “early foci”^40^ (Fig. 1a). These foci develop into larger aggregates, although not always into rings. Instead, we observed that these foci could develop into 4 different structures: rings, lines, a single large focus, or multiple larger foci. Careful analysis of time-lapse recordings for each of these 92 cells revealed evidence to suggest that distinct pathways gave rise to each of these four types of structures.

**Figure 2:**
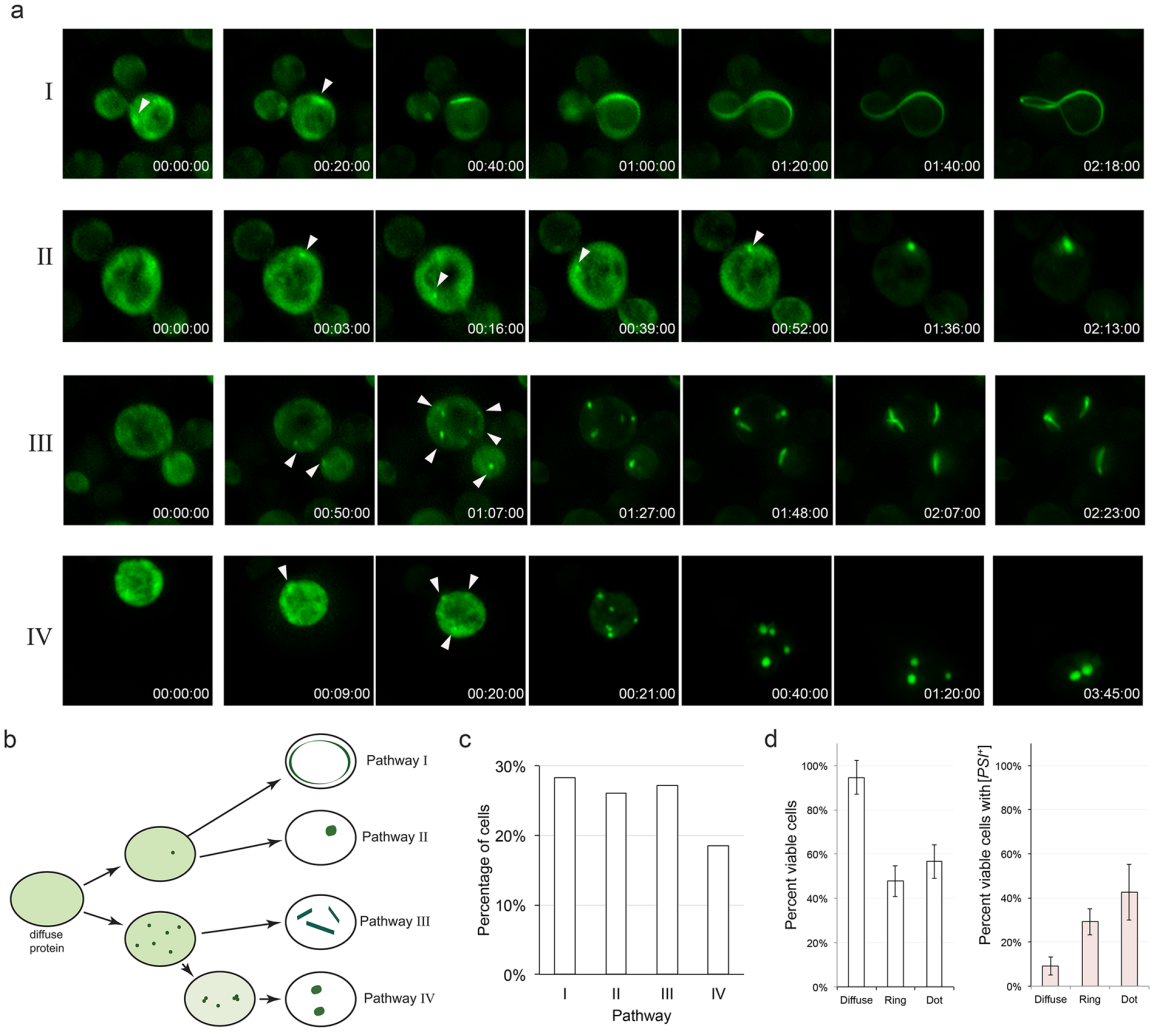
4D live cell fluorescent imaging indicates that there are four major pathways to fluorescent structures. (**a**) Diffuse cells were imaged over time after 18 hours of Sup35PrD-GFP overexpression. Representative images of four distinct pathways are shown: (I) a single focus developing into a ring, (II) a single focus developing into a dot, (III) multiple foci developing into multiple lines, and (IV) multiple foci developing into multiple dots. The time indicated is relative to foci appearance in HH:MM:SS. Arrowheads indicate the emergence of an early focus or foci. (**b**) Diagrammatic representation of the four pathways. (**c**) Ninety-two time-lapsed cells were categorized into the four pathways. Statistical analysis using the Kolmogorov Smirnov (KS) test indicated that there is no preference in pathway. (**d**) Diffuse, ring, or dot containing cells were isolated by micromanipulation and placed on rich media. Viable cells were assayed for microcolony growth on agar slabs placed on rich media (left panel). Viable colonies were streaked on rich media to assay for the presence of [*PSI* ^+^]. Standard deviation is shown.

Two of the four pathways begin with a single early focus. This single early focus can mature into a ring (pathway I) or into a larger focus (pathway II; Fig. 1b and 2a, Supplemental Fig. S1 and S2). We observed that growth into the single ring happens in two directions, which is consistent with fiber formation *in vitro* (Fig. 1b)^48^. Close inspection of dot formation indicates that the early foci grow in the X, Y, and Z planes resulting in a larger dot over time, suggesting that the dot structures grow in multiple directions (Fig. 1c).

The other two pathways involved the simultaneous formation of several small Sup35PrD-GFP foci. Similar to the single focus, multiple foci also enter into one of two pathways to eventually form several line-like structures (pathway III) or several larger dots (pathway IV; Fig. 2; Supplemental Fig. S3 and S4). Similar to growth of the ring, the lines appear to grow in two directions from either end, whereas dot structures appeared to thicken over time (data not shown). Based on our observations, a single small focus or several small foci can enter either a ring/line pathway or a dot-forming pathway (pathway I/II vs. pathway III/IV; Fig. 2b). Single early foci never formed multiple lines or dots, however, we did observe that multiple early foci could coalesce to form a single line or focus (Supplemental Fig. S4).

Of the ninety-two time-lapsed cells, the number of cells observed following each independent pathway was approximately equal (Fig. 2c). To confirm that these aggregate containing cells were indeed able to propagate [*PSI* ^+^], we isolated individual dot, ring or diffuse cells from 18-hour induced cultures by micromanipulation. As previously reported, ring cells had less viability than cells with diffuse fluorescence^45^. We found that dot containing cells also had reduced viability. Importantly, both ring and dot containing cells were able to form [*PSI* ^+^] colonies (Fig. 2d).

#### Early foci form preferentially in G2/M cells

**Figure 3:**
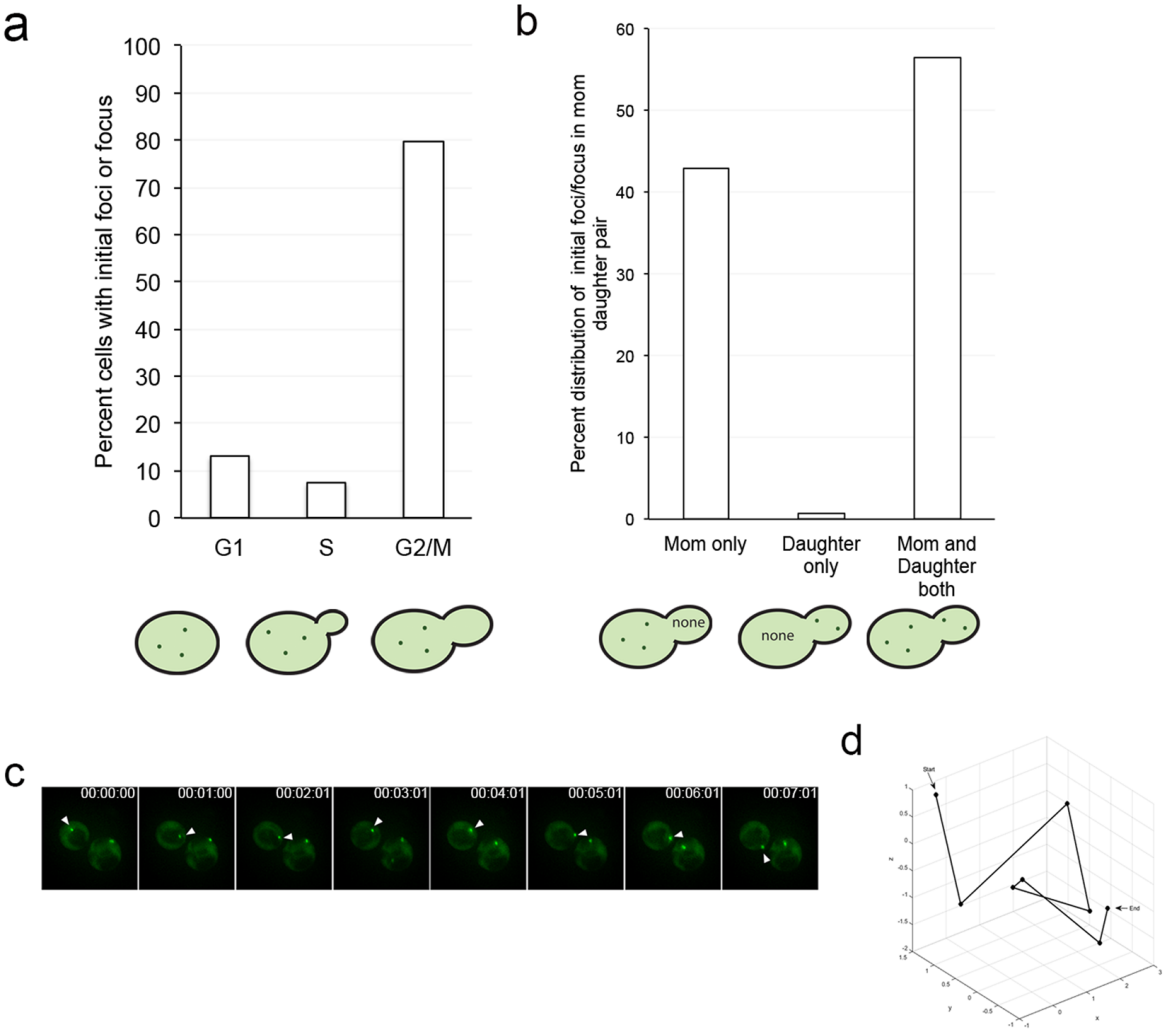
Early foci can be highly mobile and form preferentially in either the mother or simultaneously in the mother and daughter. (**a**) Early foci appearance and the stage of the cell cycle were assessed in 54 cells. (**b**) In a separate experiment, early foci were screened in 243 G2/M phase cells to determine whether the foci initially appeared in the mother cell, the daughter bud, or in the both the mother cell and daughter bud. (**c**) Time-lapse of a highly mobile early focus (arrow indicates a specific focus over time). (**d**) 3D coordinates of the focus in C were measured in the coordinates of the X, Y, and Z planes, and used to generate a 3D plot depicting the mobility of foci. The particle trajectory is indicated from start to end.

Our microscopy revealed new insights into the timing and behavior of early foci. We noticed that the early foci tended to appear in cells that were in the G2/M stage (Fig. 3a). The early foci preferentially appeared in the mother cell, or simultaneously in both the mother cell and daughter bud (Fig. 3b). Early foci either showed limited movement or exhibited high mobility (Fig. 3c,d). Those that exhibited large movements became more static over time (Supplementary Fig. S4). The transmission of aggregates from mother to daughter cell was very rare to observe microscopically, possibly due to speed and size of the transmitted particles.

### Newly formed SDS-resistant oligomer migration changes during induction

**Figure 4:**
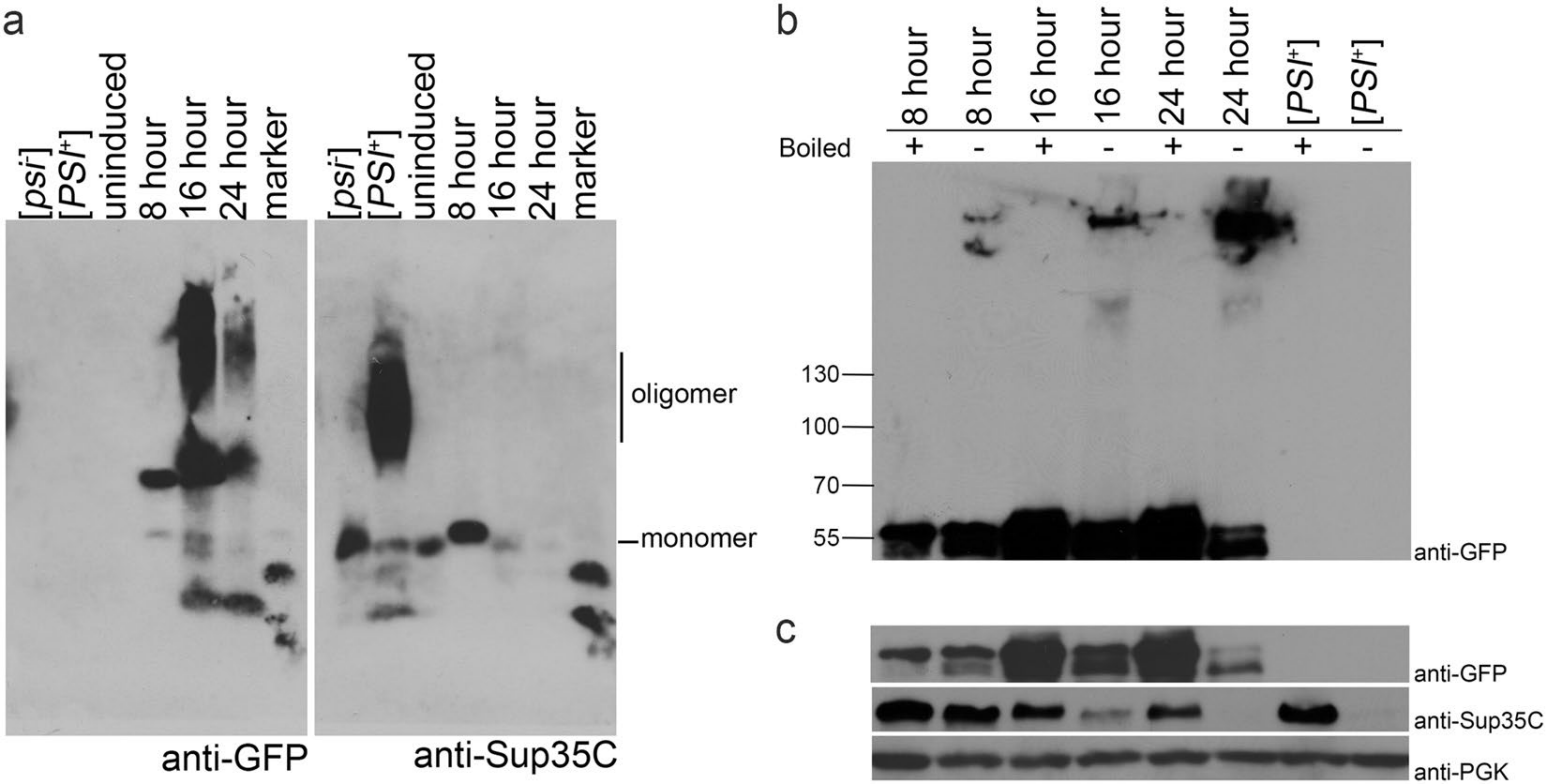
Sup35PrD-GFP and endogenous Sup35 SDS-resistant oligomers show change in migration over time. (**a**) Sup35PrD-GFP was over expressed for 8, 16 and 24 hours. Cultures were lysed and immediately subjected to SDD-AGE-immunoblot. [*psi* ^-^], established [*PSI* ^+^], and uninduced strains carrying the Sup35PrD-GFP plasmid were run as controls. Samples were run on the same agarose gel and separated for immunoblotting. The blots were incubated with either anti-GFP (left) or anti-Sup35C antibody (right). Anti-GFP antibody exclusively labels the overproduced Sup35PrD-GFP protein whereas Sup35C antibody exclusively labels the endogenous Sup35p protein. Blots were aligned in the figure based on the migration of bands in the marker lane. The migration of the monomeric endogenous Sup35p band from [*psi* ^−^] cultures and the oligomeric smear from [*PSI* ^+^] cultures is indicated on the right. While all cultures were lysed and immediately loaded onto SDD-AGE, the [*PSI* ^+^] control was stored for four days at −80 °C, which likely explains the minor degradation products. Replicate blots showed that fresh [*PSI* ^+^] lysates lacked any degradation products. (**b**) Similarly induced cultures were boiled or unboiled and run on a 7% SDS PAGE, and immunoblotted with anti-GFP antibody. The molecular weights (kDa) of the marker is as indicated on the left. Monomeric Sup35PrD-GFP migrates at approximately 55 kDa. (**c**) The top panel is a shorter exposure of the same blot from B (anti-GFP), showing the monomeric Sup35PrD-GFP bands. The same blot was re-immunolabeled with anti-Sup35 (middle) and anti-PGK (bottom) as indicated.

Since we observed visual changes in fluorescent structures over time, we next asked whether this is reflected in the formation of SDS resistant oligomers over time. Sup35PrD-GFP was induced for varying times and lysates were subjected to SDD-AGE analysis^49^. Biochemical analysis of 8-hour cultures exhibited a Sup35PrD-GFP band that migrates slightly higher than the Sup35p monomer found in [*psi* ^−^] cultures on SDD-AGE analysis (Fig. 4a). While it is unclear whether this anomalously migrating band is monomeric or oligomeric, similar unboiled 8-hour lysates run on SDS-PAGE and immunoblotted showed high molecular weight species migrating near the top of the gel (Fig. 4b). Since fluorescent early foci, dots, and rings begin to appear between 12–16 hours of induction (data not shown), it is likely that Sup35PrD-GFP begins to assemble into larger species before the visualization of fluorescent aggregates. 16-hour cultures run on SDD-AGE showed a bipartite smear that suggests that Sup35PrD-GFP undergoes further assembly into larger oligomeric species, although it is unclear whether these bands are composed of Sup35PrD-GFP alone or a complex of other proteins. By 24 hours, some of the slowest migrating oligomeric species disappear, suggesting that these absent oligomers may be degraded, or are unresolvable between 16 and 24 hours. Induced lysates were also run on SDS-PAGE with or without boiling. As expected, we observed that the levels of overexpressed Sup35PrD-GFP in boiled samples increased with time of induction, as well as the increasing accumulation of Sup35PrD-GFP near the top of the gel over time (Fig. 4b and c). It is also noted that monomeric Sup35PrD-GFP in unboiled samples loses intensity over time (Fig. 4c), suggesting that the majority of overexpressed protein is in high molecular weight assemblies at 24 hours.

### Endogenous Sup35p forms SDS-resistant oligomers during induction

The assembly of endogenous Sup35p into high molecular weight SDS-resistant oligomers on SDD-AGE gels was only apparent upon longer exposures of Western blots (Supplemental Fig. S5). These SDS-resistant oligomers co-migrated with Sup35PrD-GFP oligomers at 24 hours. To confirm that endogenous Sup35p was assembling into SDS-resistant species, we looked at endogenous Sup35p levels in induced cultures by SDS-PAGE. While 8-hour unboiled lysates showed a substantial amount of endogenous Sup35p, bands were fainter in 16-hour unboiled cultures, and absent in 24-hour unboiled samples (Fig. 4c). The lack of readily detectable endogenous Sup35p polymers at 24 hours on SDD-AGE (Fig. 4a), yet loss of solubility on SDS-PAGE (Fig. 4c) suggests that Sup35p is possibly assembling into extremely large SDS-resistant molecular weight species that cannot be resolved by our gel systems.

### Newly formed prion particles are infectious

Several studies have previously shown that lysates from established [*PSI* ^+^] cultures and *in vitro* assembled aggregates are infectious^21, 22, 50^. Our biochemical studies showed that high molecular weight SDS resistant species begin to appear at 8 hours and persist over time (Fig. 4b). We asked whether lysates containing these new high molecular weight species were infectious, meaning that the lysate alone is able to convert [*psi* ^−^] cells to the [*PSI* ^+^] state^40, 47^.

**Figure 5:**
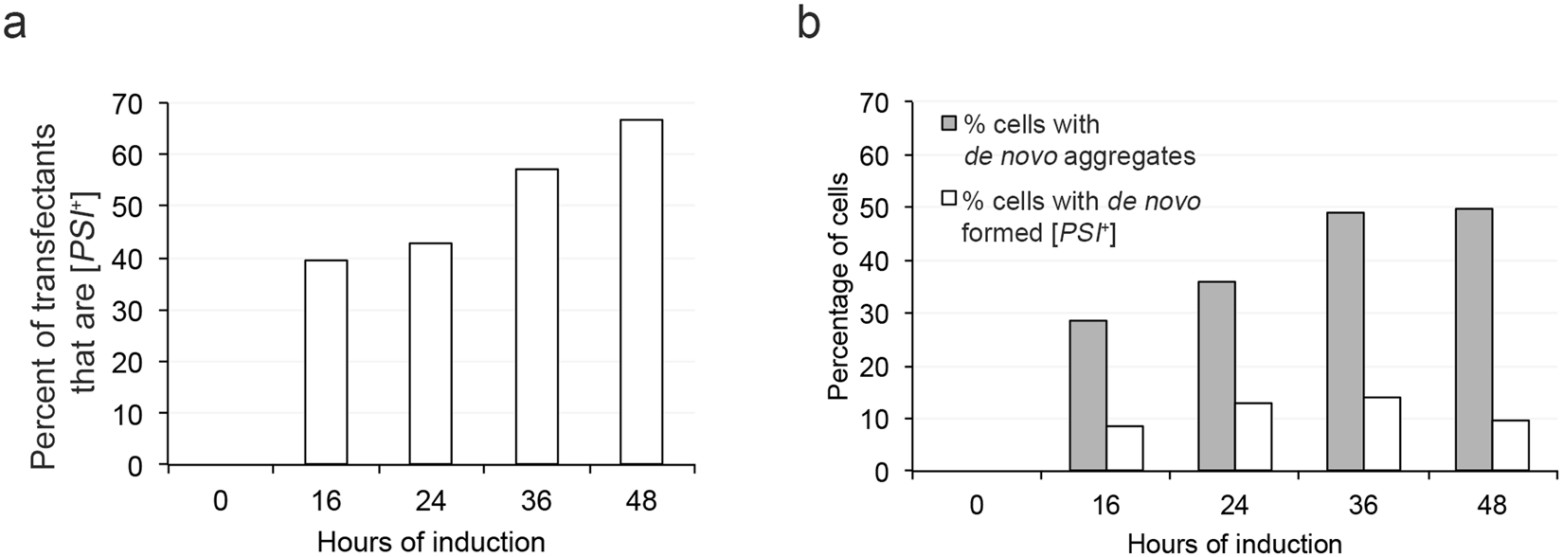
*De novo* induced prion particles are infectious. (**a**) Donor lysates from cultures induced for the indicated times (x-axis) were transfected into [*psi* ^−^][*PIN* ^+^] cultures. Transfection conversion frequencies are shown. (**b**) The same donor cultures, used in transfection, were also assessed for the ability to form Sup35PrD-GFP aggregates (rings, lines, and dots; grey bars) and *de novo* induction frequency (white bars). This data represents single trial out of three independent trials.

We performed transfection experiments using lysates induced for 8, 16, 24, 36 and 48 hours. We verified that these cultures formed dot and ring/line aggregates (Fig. 5b). *De novo* induced cell cultures were lysed and transfected into [*psi* ^−^][*pin* ^−^] and [*psi* ^−^][*PIN* ^+^] recipient cells. Crude lysates were immediately mixed with the spheroplasted recipients for transfection to limit additional *in vitro* aggregation. Transfectants were scored for the conversion to [*PSI* ^+^] using a color assay (see materials and methods). Consistent with previously published results, control [*PSI* ^+^] lysates were able to convert [*psi* ^−^][*PIN* ^+^] and [*psi* ^−^][*pin* ^−^] recipient cells to [*PSI* ^+^]^21, 22^ (Table 1). The obtained [*PSI* ^+^] transfectants were due to conversion and not from contamination of whole-cells in the crude extracts given that no viable cells were detected when lysates were directly plated on rich media. We also distinguished all true [*PSI* ^+^] colonies from *ADE1* suppressor mutations by curing on media containing 5 mM guanidine hydrochloride^51^.

**Table 1:** Transfection of *de novo* formed Sup35PrD-GFP oligomers convert [*psi* ^−^] into [*PSI* ^+^].

	Control lysates		Lysates prepared after Sup35PrD-GFP overexpression for				
	[*psi* ^−^]	[*PSI* ^+^]	16 hrs [*psi* ^−^] [*PIN* ^+^]	24 hrs [*PIN* ^+^] [*psi* ^−^]	36 hrs [*PIN* ^+^] [*psi* ^−^]	48 hrs [*PIN* ^+^] [*psi* ^−^]	48 hrs [*pin* ^−^] [*psi* ^−^]
Recipient [*psi* ^−^] [*PIN* ^+^]	0%	30.37%	39.5%	42.9%	56.9%	66.6%	0%
Recipient [*psi* ^−^][*pin* ^−^]	0%	17.5%	ND	ND	ND	51.7%	0%

Lysates from control strains ([*psi* ^−^] or [*PSI* ^+^]) or strains overexpressing Sup35PrD-GFP for indicated times were transfected into [*psi* ^−^][*PIN* ^+^] or [*psi* ^−^][*pin* ^−^] recipient cultures. Numbers indicate the percent of transfectants (approximately 200–300 transfectants scored) that were [*PSI* ^+^]. ND = not determined. This table represents data from a single trial out of three independent trials. A binomial comparison test was performed between [*psi* ^−^] and either [*PSI* ^+^] or the induced cultures values, in order to determine the success of each pairing. All p-values obtained per pair in each of the three trials were less than 0.03. Simple linear regression indicated that transfection frequencies between 16, 24, and 48 hours were not different for all three trials.

Transfection of lysates obtained from cultures overexpressing Sup35PrD-GFP for 8 hours showed no [*PSI* ^+^] conversion, indicative of zero infectivity (data not shown). Therefore, the initial appearance of a high molecular species in 8-hour cultures on SDS-PAGE (Fig. 4b) does not correlate with infectivity. Surprisingly, 16 hour induced lysates lead to 39.5% of transfected cells being converted to [*PSI* ^+^]. These conversion frequencies increased over time, with 48-hour induced cultures leading to 66% conversion (Table 1, Fig. 5a). We found that infectivity is correlated with the presence of fluorescent structures, and not only the elevated level of Sup35PrD-GFP protein, since lysates from [*psi* ^−^][*pin* ^−^] cells overexpressing Sup35PrD-GFP for 48 hours, which do not form fluorescent aggregates, failed to convert recipient cells to [*PSI* ^+^] (Table 1). As a whole, all induced cultures, except 8-hours, yielded prion conversion comparable to the established [*PSI* ^+^] lysate control^36^. We observed that two different structural variants of [*PSI* ^+^], weak and strong, were equally distributed among recipients receiving 16 and 24 hour induced lysates^21^ (Supplemental Fig. S6).

We wanted to determine whether transfection frequencies were similar to [*PSI* ^+^] that was formed *in vivo.* The same induced cultures that were used in the transfection experiment were also plated and scored for their ability to form [*PSI* ^+^]. We observed that *in vivo* formation of [*PSI* ^+^] through induction was considerably less than the conversion by transfection (Fig. 5b). Furthermore, we asked whether [*PIN* ^+^] was a requirement for conversion to [*PSI* ^+^] during transfection. [*PIN* ^+^] has been shown to be important for the cross seeding of Sup35p into the prion form^35, 36, 38, 40^. When 48-hour lysates were transfected into [*PIN* ^+^] cells or [*pin* ^−^] cells, similar levels of conversion were observed (Table 1). Taken together, this data confirms that [*PIN* ^+^] is required for initial cross seeding but is dispensable for the transfection mediated conversion of [*PSI* ^+^].

## Discussion

The formation of aberrant protein assemblies is associated with a vast array of human diseases, including prion disease and several neurodegenerative diseases. Numerous studies have shed light on the initial formation of macromolecular aggregates *in vitro,* yet less is known about *in vivo* formation since capturing these rare events is extremely challenging. Our time-lapse data show that fluorescent structures form during *de novo* induction through four separate pathways (Fig. 2). Different sized SDS resistant oligomers are associated with various stages of induction (Fig. 4). We found that lysates associated with these different stages of induction were as infectious as lysates from cultures with established [*PSI* ^+^].

All 92 cells visualized by time-lapse recordings started with the formation of early foci. While cytosolic inclusions such as the insoluble protein deposit (IPOD) have been suggested to be associated with initial prion formation^47^, it is unclear whether these static foci reside in IPOD. We show that early foci grow bidirectionally in pathways I and III, to form rings and lines (Fig. 1b). This is consistent with the bidirectional growth of recombinant Sup35PrD seen *in vitro* ^48^. The growth of large dot structures, on the other hand, appears to expand in three dimensions (Fig. 1c), suggesting that the mechanism of assembly of rings and dots is different. Cryo-electron microscopy experiments previously showed ring structures are composed of bundles of uninterrupted long fibrils whereas dot structures are composed of short fibril bundle structures^47^. Taken together, the inherent conformation of the initial foci may determine whether it is going to follow ring versus dot pathway, both of which are able to give rise to [*PSI* ^+^] progeny.

Our data showed distinct changes in the migration of SDS-resistant oligomers during *de novo* induction. The initial appearance of Sup35PrD-GFP SDS-resistant species begins at 8-hours (Fig. 4b), which is detected before fluorescent foci become visible. This data corroborates our assumption that protein misfolding begins much earlier than microscopic visualization of fluorescent foci. The SDD-AGE profiles of *de novo* induced cultures at later time points show two major migrating smears. It is unclear how this bipartite smearing pattern corresponds with [*PSI* ^+^] induction. It may represent different populations of cells, such as those with dots or rings, or include both transmissible and non-transmissible oligomers (Fig. 4a). Even with the variety of structures formed, levels of soluble endogenous Sup35p appear to diminish over time (Fig. 4c). This loss in solubility suggests that endogenous Sup35p assembles into larger SDS-resistant oligomers. This difference in the migration between SDS-resistant endogenous Sup35p at 24 hours and that of established [*PSI* ^+^] (Supplemental Fig. S5) could indicate that newly formed oligomers undergo further structural changes to become established as a propagating prion.

We asked whether the newly formed prion particles are infectious. Our data showed that transfection of lysates containing both high molecular weight SDS-resistant Sup35PrD-GFP species and insoluble endogenous Sup35p (16 and 24 hours) was able to convert [*psi* ^−^] recipient cells to [*PSI* ^+^]. We also noticed a difference between the *in vivo* [*PSI* ^+^] induction frequency, and the conversion frequency of [*PSI* ^+^] through transfection (Fig. 5). This discrepancy suggests that the oligomers themselves are infectious but potential barriers, such as quality control mechanisms, may exist *in vivo* to reduce their transmission and propagation. Several studies have shown that modulation of chaperones, autophagy, the ubiquitin-proteasome system, and factors associated with the cytoskeleton can alter the *de novo* induction of [*PSI* ^+^]^30, 45, 52–59^. Therefore, activation of the stress response and other cellular mechanisms during Sup35PrD-GFP overexpression could explain why we observed a remarkable difference between *in vivo* formed induction frequencies and transfection conversion frequencies. It can be envisioned that the release of prion propagons during lysis liberates these particles from the control of protein quality control machinery, which would contribute to their increased infectivity. Conversely, “naïve cells” whose quality control systems are not as active as those experiencing Sup35PrD-GFP overexpression, could be more susceptible to prion conversion by the introduction of newly formed oligomers. Another explanation could be that spatial quality control mechanisms are at play *in vivo*. We showed that the initially formed foci preferentially appear in cells that are in G2/M phase. It has been shown that chaperones, such as Hsp104p, asymmetrically distribute to the mother cell during recovery from stress in order to retain damaged proteins while leaving the daughter rejuvenated^60–62^. It is possible that under induction conditions, the redistribution of chaperones before mitosis may make the cell vulnerable to the formation of the observed fluorescent structures. However, since spatial quality control mechanisms likely clear the daughter cell of damaged or misfolded proteins, it is also possible that similar mechanisms may free the daughter cells from efficiently inheriting newly formed oligomers *in vivo*.

The formation of protein aggregates is a common feature in many neurodegenerative and amyloid diseases. Yet, underlying mechanisms behind how these aggregates form and their infective qualities are poorly understood. Our study shows that the formation of newly formed prion particles is more complex than previously thought. Prion particles can form through multiple pathways yet hold inherent infectivity. Our studies of [*PSI* ^+^] formation suggest that assembly and remodeling of newly formed endogenous oligomers may be required to establish a propagating amyloid. Yet, our novel findings suggest both established oligomers and newly formed oligomers are fundamentally infective.

## Materials and Methods

### Yeast strains, plasmids and growth conditions

The [*PSI* ^+^][*pin* ^−^] and [*psi* ^−^][*PIN* ^+^] strains used in this study were derivatives of 74-D694 (*MATa ade1–14 leu2-3, 112 his3-Δ200 trp1-289 ura3-52*; D233)^34^. [*PIN* ^+^] strains used were of ‘high’ variant type^63^. Plasmid p3032, which was used to induce [*PSI* ^+^] *de novo*, is a low-copy centromeric plasmid containing the selectable marker LEU2, and the PrD region of Sup35p (1–254) fused to GFP under the *CUP1* promoter (SUP35PrD-GFP^44^). Strains transformed with this plasmid were grown on media lacking leucine (-Leu). p3116 (pRS316) with the selectable marker URA3 was used as a empty vector for transfection experiments^64^. *Saccharomyces cerevisiae* strains were grown at 30 °C using standard media and cultivation procedures^65^. Rich media contained 2% dextrose (YPD) or 2% glycerol (YPG). Curing of strains of prions was performed on rich media supplemented with 5 mM GuHCl. Synthetic media (SD) contained 2% dextrose and appropriate amino acids. Lithium acetate method was used for yeast transformations^66^.

### [*PSI*^+^] color assay

All yeast strains used in our study have the *ade1-14* allele that has a nonsense mutation and enables the scoring of [*PSI* ^+^]. In [*psi* ^−^] cells, the Sup35p translation termination factor is soluble and prematurely terminates Ade1p synthesis. These cells accumulate red pigment (a precursor in the adenine synthesis pathway) in rich media like YPD, and fail to grow on media lacking adenine (SD-Ade)^67^. However in [*PSI* ^+^] cells, the majority of Sup35p is aggregated and unavailable for translation termination, resulting in the read through of *ade1-14* premature stop codon. The synthesis of full length Ade1 protein results in non-red colonies on rich media and growth on SD-Ade. The variant weak [*PSI* ^+^] enables a small amount of readthrough such that colonies appear pink, whereas strong [*PSI* ^+^] facilitates more substantive readthrough such that colonies appear white. [*PSI* ^+^] cells were distinguished from nonsense suppressor mutations by curing on GuHCl.

### Time-lapse fluorescent microscopy and micromanipulation

Cells in this study were visualized by using a Leica DMI 6000 deconvolution microscope (63 X oil immersion with N.A 1.4 magnifier) with camera DFC365FX. Aggregates were visualized using the GFP and DIC channels. Leica LASX software was used to capture approximately ten Z-stacks layers (set to approximately 0.5–1 µm increments) for time-lapse images of cells every 30 sec or 1 minute for twelve hours. Images were subjected to 3D deconvolution using Autoquant deconvolution algorithms (Media Cybernetics).

For time-lapse experiments, 74-D694 [*psi* ^−^][*PIN* ^+^] cells were transformed with p3032 (Sup35PrD-GFP) and grown overnight in SD –Leu medium containing 25 μM CuSO_4_. 5 μl of cells were then added in 295 μl of fresh SD –Leu media to Ibidi 1 μ-slide 8 well glass bottom slides (Ibidi USA, Inc. Madison, WI). Glass slides were treated with concanavalin A before adding cells, to ensure cells would adhere to the slide surface. Cells were well mixed and allowed to settle for 5 minutes. Time-lapse movie clips were acquired from randomly chosen diffuse cells for the time described. To plot the movement of early foci, X, Y, and Z coordinates were obtained using the Leica LASX software. The coordinates were imported into MATLAB and a three-dimensional plot was created.

Cytoplasmically diffuse, ring or dot containing cells were micromanipulated under fluorescent microscopy using a Zeiss Axiovert 200 fluorescent microscope from cultures that were induced for approximately 18 to 22 hours. Cells were dissected onto 1% noble agars slabs. Slabs were then placed on rich media without copper, to limit Sup35PrD-GFP overexpression. Microcolonies were streaked on rich media to observe color. Non-red colonies were cured by streaking several times on media containing 5 mM GuHCl to rule out the presence of nonsense suppressors^68^.

### Biochemical analysis of yeast lysates

Cell lysates were prepared from 50 ml of overnight cultures. Cells were harvested and lysed in the presence of lysis buffer (80 mM Tris, 300 mM KCl, 10 mM MgCl_2_, 20% [wt/vol] glycerol, 1:50 diluted protease inhibitor cocktail [Sigma], and 5 mM PMSF) at pH 7.6. 0.5 mm glass beads (Biospec Inc.) were added and vortexed at high speed for 1 min followed by 1 min cooling in ice. This was repeated 5 times. Lysates were precleared of cell debris by centrifuging two times at 600 g for 1 min at 4 °C^69^.

To analyze [*PSI* ^+^] aggregates by SDD-AGE, ~60 μg of freshly made crude lysate was treated with 2% SDS sample buffer (25 mM Tris, 200 mM glycine, 5% glycerol, and 0.025% bromophenol blue), incubated for 7 min at room temperature, electrophoretically resolved in a horizontal 1.5% agarose gel in a standard tris/glycine/SDS buffer, transferred to a polyvinylidene difluoride (PVDF) membrane, and immunoblotted with anti-GFP or anti-Sup35C antibody as described previously^49^.

For SDS-PAGE analysis ~100 μg of crude lysate was treated with 2% SDS sample buffer and was boiled or unboiled as indicated^70^. Samples were run on 7% gels and subjected to standard western blot procedures. The PageRuler^TM^ Prestained Protein Ladder (Cat# 26616, Thermo Fisher Scientific) was used on SDS-PAGE gels.

### Transfection

74-D694 [*PIN* ^+^] or [*pin* ^−^] strains were induced with copper for the indicated times and lysed using standard procedures^71^. Transfection of freshly made donor induced lysates was performed using a previously published protocol^71^. To confirm that donor lysates were free of viable cells, lysates were plated on YPD. Only lysates that did not have live cell contamination were counted as transfection experiments to be analyzed. Recipient [*PIN* ^+^] or [*pin* ^−^] strains were grown to mid log phase and converted into spheroplasts by lyticase treatment. Spheroplast populations were mixed with the indicated freshly made donor lysates and an empty vector (pRS316) to aid in more efficient transfection. This mixture was plated on medium that selects for plasmid (Sorbitol-Ura). Transformants that grew on Sorbitol-Ura were screened for their [*PSI* ^+^] status by assaying pink and white colony color on complete media. Furthermore, colonies were cured on 5 mM GuHCl in order to eliminate the possibility of nonsense suppressor mutations^51, 68^. To determine the success of transfectant values between [*psi* ^−^] and either [*PSI* ^+^] controls or the induced cultures, a binomial comparison was performed for each pairing. Simple linear regression was performed to determine if transfection percentages obtained between 16, 24, and 48-hour lysates in one trial were different.

## Electronic supplementary material

Supplementary information

Supplemental Figure 1

Supplemental Figure 2

Supplemental Figure 3

Supplemental Figure 4
