# Supplementary material for: De novo [PSI+] prion formation involves multiple pathways to form infectious oligomers
Source: Sci Rep. 2017 Mar 6;7:76. doi: 10.1038/s41598-017-00135-6 (PMC5427932; doi:10.1038/s41598-017-00135-6)
Supplement: Supplementary file 1 — Supplementary information [file 41598_2017_135_MOESM1_ESM.pdf]

Title: *De novo* [*PSI*$^+$] prion formation involves multiple pathways to form infectious oligomers

Authors: Jaya Sharma[1], Brett Wisniewski[1], Emily Paulson[2], Joanna Obaoye[1], Stephen J. Merrill[2] and Anita L. Manogaran[1*]

Affiliation: [1]Department of Biological Sciences,

Marquette University. Milwaukee, WI 53201

[2] Department of Mathematics, Statistics and Computer Science, Marquette University, Milwaukee, WI 53201

*Corresponding author: Anita L. Manogaran

Current mailing address:

Department of Biological Sciences

P.O. Box 1881

Marquette University

Milwaukee, WI 53201-1881

Office: (414) 288-4580

Email: anita.manogaran@marquette.edu

Supplementary Information Figure Legends

**Supplementary Figure 1. Video - 4D Live cell imaging of the pathway I during *de novo* prion induction.** Timelapse movies of the four cells shown in figure 2a. Movies are 10 frames per second, with each second representing 4 minutes and 30 seconds of time. Movie images were captured in 3D, deconvolved and collapsed.

**Supplementary Figure 2. Video - 4D Live cell imaging of the pathway II during *de novo* prion induction.**

**Supplementary Figure 3. Video - 4D Live cell imaging of the pathway III during *de novo* prion induction.**

**Supplementary Figure 4. Video - 4D Live cell imaging of the pathway IV during *de novo* prion induction.**

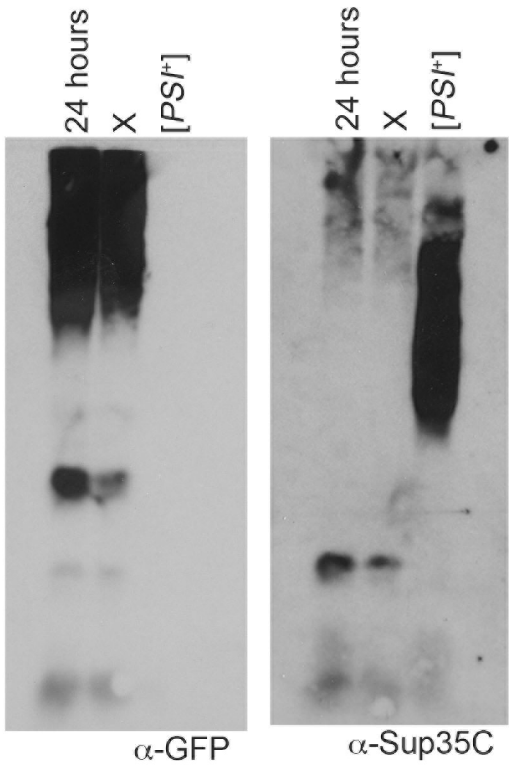


**Supplementary Figure 5. Overexpressed Sup35PrD-GFP and endogenous Sup35p co-migrate as high molecular weight assemblies in culture induced for 24 hours.** Lysates from 24 hour induced cultures in a [*psi*$^-$][*PIN*$^+$] background were treated with SDS without boiling. Samples were run on the same SDD-AGE gel (approximately 2%) and separated for immunoblotting. The blots were incubated with either anti-GFP (left) or anti-Sup35C antibody (right). Lysates from [*PSI*$^+$] cultures were run as a control. X indicates a sample unrelated to this study.

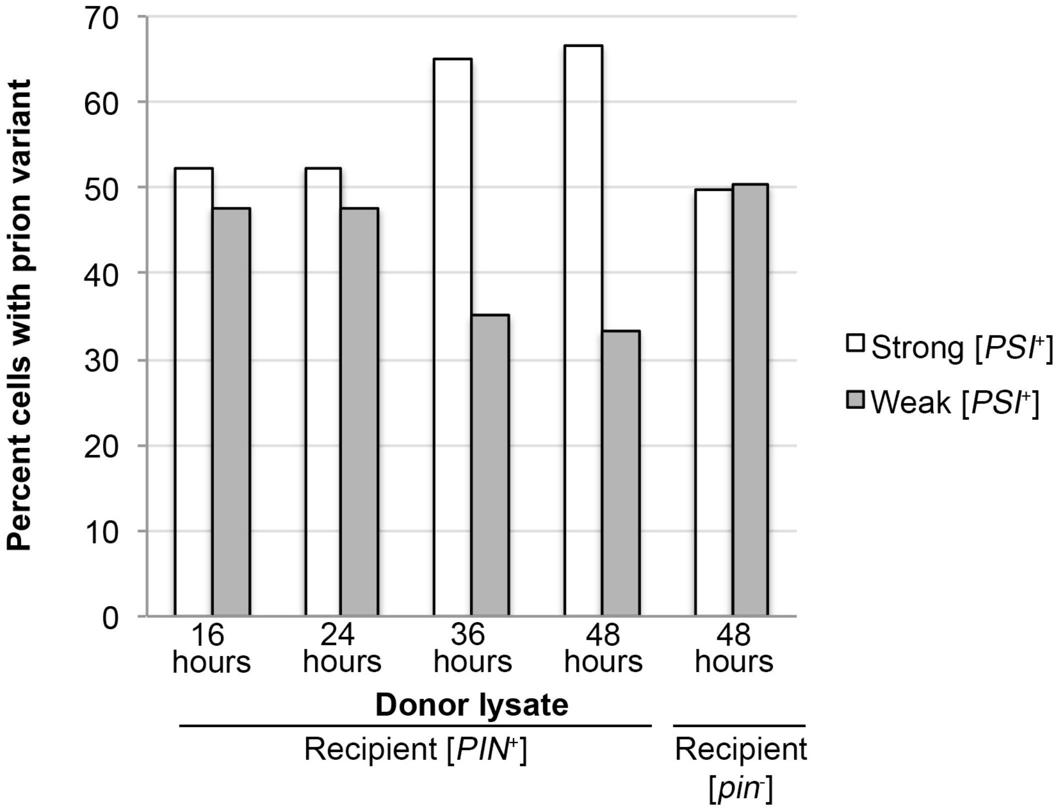


**Supplementary Figure 6. Weak [*PSI*$^{+}$] variants predominate over strong [*PSI*$^{+}$] variants when transfected with lysates from cultures with long Sup35PD-GFP overexpression times.** Cells transfected with lysates from figure 5A were further scored for the presence of strong (white bars) and weak (gray bars) [*PSI*$^{+}$].
